# Psychosis and Schizophrenia-Spectrum Personality Disorders Require Early Detection on Different Symptom Dimensions

**DOI:** 10.3389/fpsyt.2019.00476

**Published:** 2019-07-11

**Authors:** Frauke Schultze-Lutter, Igor Nenadic, Phillip Grant

**Affiliations:** ^1^Department of Psychiatry and Psychotherapy, Medical Faculty, Heinrich-Heine-University, Düsseldorf, Germany; ^2^Department of Psychiatry and Psychotherapy, Philipps-Universität Marburg/UKGM, Marburg, Germany; ^3^Psychology School, Faculty of Health and Social Sciences, Fresenius University of Applied Sciences, Frankfurt am Main, Germany; ^4^Faculty of Life Science Engineering, Technische Hochschule Mittelhessen University of Applied Sciences, Giessen, Germany; ^5^Department of Biological Psychology and Individual Differences, Justus-Liebig-University, Giessen, Germany

**Keywords:** psychosis, schizotypy, schizotypal personality disorder, prediction, positive dimension, negative dimension, disorganized dimension

## Abstract

Psychotic disorders and schizophrenia-spectrum personality disorders (PD) with psychotic/psychotic-like symptoms are considerably linked both historically and phenomenologically. In particular with regard to schizotypal and schizotypal personality disorder (SPD), this is evidenced by their placement in a joint diagnostic category of non-affective psychoses in the InternationaI Classification of Diseases 10th Revision, (CD-10) and, half-heartedly, the fifth edition of Diagnostic and Statistical Manual of Mental Disorders, (DSM-5). Historically, this close link resulted from observations of peculiarities that resembled subthreshold features of psychosis in the (premorbid) personality of schizophrenia patients and their biological relatives. These personality organizations were therefore called “borderline (schizophrenia)” in the first half of the 20th century. In the 1970s, they were renamed to “schizotypal” and separated from psychotic disorders on axis-I and from other PD on axis-II, including modern borderline PD, in the DSM. The phenomenological and historical overlap, however, has led to the common assumption that the main difference between psychotic disorders and SPD in particular was mainly one of severity or trajectory, with SPD representing a latent form of schizophrenia and/or a precursor of psychosis. Thus, psychosis proneness and schizotypy are often assessed using SPD questionnaires. In this perspective-piece, we revisit these assumptions in light of recent evidence. We conclude that schizotypy, SPD (and other schizophrenia-spectrum PD) and psychotic disorder are not merely states of different severity on one common but on qualitatively different dimensions, with the negative dimension being predictive of SPD and the positive of psychosis. Consequently, in light of the merits of early diagnosis, the differential early detection of incipient psychosis and schizophrenia-spectrum PD should be guided by the assessment of different schizotypy dimensions.

The group of psychotic disorders mainly includes non-affective (i.e., schizophrenia and schizophrenia-spectrum psychoses) and affective psychoses (i.e., mania, bipolar disorders, and depression) whose common features are positive psychotic symptoms (i.e., delusions and hallucinations) (1). Personality disorders (PD) with positive and negative psychotic-like features are assumed to be closely related to the schizophrenia spectrum; these are paranoid PD, schizoid PD, and schizotypal PD (SPD).

Despite their low lifetime prevalence of about 2% (1, 2), psychoses cause tremendous costs, burden, and disability, already in children and adolescents (3–5). Because a long duration of nontreatment of psychosis and its prodrome negatively impacts outcome (6), research on an early detection and intervention in psychosis prior to the first episode increasingly gained momentum since the 1990s. By now, clinical high-risk (CHR) criteria have already been suggested for transfer into clinical practice, e.g., within the framework of the guidance project of the European Psychiatric Association (7, 8).

The prevention of schizophrenia-spectrum PD is less clear. In the United States, the lifetime prevalence of schizophrenia-spectrum PD was 9% in adults of age ≥20; with SPD 3.9%, paranoid PD 4.3%, and schizoid PD 3.1% (9). Lower rates were reported from Norway (10) (paranoid, 2.4%; schizoid, 1.7%; SPD, 0.6%) and Germany (11) (paranoid, 1.8%; schizoid, 0.4%; SPD, 0.7%) with a higher SPD prevalence in relatives of schizophrenia patients (2.1%) (12). Little is known about the costs and burden of schizophrenia-spectrum PD beyond their assumed role of increasing risk for schizophrenia, as they are frequently not assessed in studies of societal impact of mental disorder (4, 5). Similarly, little research has specifically targeted their early detection and prevention beyond being a by-product of, e.g., research on early detection of psychosis (13).

## Personality Traits and Disorders, and Psychosis

Psychoses and schizophrenia-spectrum PD, particularly SPD, are linked historically, phenomenologically, and through shared genetic and (neuro-)biological factors (14). This link is mirrored by SPD’s placement within the ICD section for schizophrenia and related disorders and its mentioning as a related disorder in the schizophrenia section of DSM-5 (15, 16). Because SPD and schizotypy—as well as other terms often used in this context such as psychotic-like experiences (17)—are not synonymous (18); in the following, we will strictly distinguish between these terms and elucidate their conceptual differences later in the manuscript (see also **Table 1**).

**Table 1:** Current operationalizations of schizotypy, schizotypal disorder according to ICD-10, SPD and other schizophrenia-spectrum PD according to DSM-5, clinical high risk (CHR) of psychosis and psychosis (15, 19–23).

	Schizotypy	Schizotypal disorder	Schizoid (s) and paranoid (p) PD	SPD	CHR ^a^	Psychosis
**General characteristic**	*Enduring personality trait, not per se considered as pathological character*	*Evolution and chronic course (alike that of a PD) with fluctuations of intensity and no definite onset (trait-state character)*	*An enduring pattern of inner experience and behavior (trait) that deviates markedly from the expectations of the individual’s culture, is pervasive and inflexible, has an onset in adolescence or early adulthood, is stable over time, and leads to distress or impairment*		*Full or at least some insight into their abnormal nature; defined onset or worsening, not part of the premorbid personality (state)*	*Defined onset, state (positive symptoms with no insight into their abnormal nature)*
**Positive factor**	Beliefs that are regarded as invalid and magical by conventional standards, but might well be shared by certain subgroups, e.g. certain esoteric or spiritual beliefs; Distortions in the perception of one’s body and/or environmental stimuli; Sensory hypersensitivity	Odd beliefs or magical thinking, influencing behavior and inconsistent with subcultural norms; Suspiciousness or paranoid ideas; Unusual perceptual experiences including somatosensory (bodily) or other illusions, depersonalization or derealization; Occasional transient quasi-psychotic episodes with intense illusions, auditory or other hallucinations, and delusion-like ideas, usually occurring without external provocation;	Suspects, without sufficient basis, that others are exploit-ting, harming, or deceiving him/her (p); Is preoccupied with unjustified doubts about the loyalty or trustworthiness of friends/associates (p); Is reluctant to confide in others because of un-warranted fear that the information will be used maliciously against him/her (p); Reads hidden demeaning or threatening meanings into benign remarks or events (p); Perceives attacks on his/her character or reputation that are not apparent to others and is quick to react angrily or to counterattack (p); Has recurrent suspicions, without justification, regarding fidelity of spouse/sexual partner (p)	Ideas of reference (excluding delusions of reference); Odd beliefs or magical thinking that influences behavior and is inconsistent with subcultural norms (e.g., superstitious-ness, belief in clairvoyance, telepathy, or “sixth sense”: in children and adolescents, bizarre fantasies or preoccupations); Suspiciousness or paranoid ideation; Unusual perceptual experiences, including bodily illusions.	P1 unusual thought content/delusional ideas; P2 suspiciousness/persecutory ideas; P3 grandiose ideas; P4 perceptual abnormalities/hallucinations; P5 disorganized communication Unstable ideas of reference Derealization; Decreased ability to discriminate between ideas and perceptions/memories; Visual/acoustic perception disturbances immediately recognized as a problem with sensory or mental processes	Delusions; i.e., firm beliefs held with full conviction that are untrue as well as contrary to a person’s educational and cultural background Hallucinations; i.e., perceptions experienced without an external stimulus
**Negative factor**	Diminished pleasure or discomfort in social or interpersonal situations; Deficits to experience pleasure in different sensory domains or discomfort from sensory stimulation; reduction in psychomotor drive; Flattened affect or reduction in emotional expressiveness; reduction in verbal expressiveness	Constricted affect (the individual appears cold and aloof); Poor rapport with others and a tendency to social withdrawal	Neither desires nor enjoys close relationships, including being part of a family (s); Almost always chooses solitary activities (s); Has little, if any, interest in having sexual experiences with another person (s); Takes pleasure in few, if any, activities (s); Lacks close friends or confidants other than first-degree relatives (s); Appears indifferent to the praise or criticism of others. Shows emotional coldness, detachment, or flattened affectivity (s)	Lack of close friends or confidants other than first-degree relatives Excessive social anxiety that does not diminish with familiarity and tends to be associated with paranoid fears rather than negative judgments about self Constricted affect.	*Not part of CHR criteria:* N1 social withdrawal; N2 avolition; N3 expression of emotion; N4 experience of emotion and self; N6 occupational functioning; D3 trouble with focus and attention. Multiple self-experienced impairments in drive, stress tolerance, affect, emotional responsiveness, desire for social contact, social skills, attention concentration, and memory	Anhedonia (in social and other activities/ situations); Avolition; Affective flattening; Reduced intensity of emotional response; Attentional impairment; Alogia
**Disorganized factor**	Speech deficits due to disorganized, confused thinking that do not cause grave problems in other people’s understanding of the person; Simultaneous experience of divergent emotions	Vague, circumstantial, metaphorical, overelaborate, or stereotyped thinking, manifested by odd speech or in other ways, without gross incoherence; Behavior or appearance that is odd, eccentric, or peculiar; Inappropriate affect		Odd thinking and speech (vague, circumstantial, metaphorical, overelaborate, or stereotyped). Behavior or appearance that is odd, eccentric, or peculiar. Inappropriate affect	*Not part of CHR criteria:* D1 odd behavior and appearance; D2 bizarre thinking; D4 impairment in personal hygiene N5 ideational richness	Formal thought disorder/disorganized speech that severely hinders other people’s understanding of the person; Disorganized or bizarre behavior; Incongruous affect
**Cognitive factor** **^b^**					Thought interference; Thought blockage; Thought pressure; Thought perseveration; Disturbances of abstract thinking; Disturbance of receptive; Disturbance of expressive speech; Inability to divide attention; Captivation of attention	
**Others**			Obsessive ruminations without inner resistance, often with dysmorphophobic, sexual or aggressive content	Persistently bears grudges (i.e., is unforgiving of insults, injuries, or slights) (p)			
**Features/symptoms needed for diagnosis**	Not applicable, no mental disorder	3 or more, each present for at least 2 years	4 or more	5 or more	1 or more (APS, BIPS, COPER criteria) 2 or more (COGDIS criterion)	Dependent on type of psychotic disorder

a According to the Structured Interview for Psychosis-Risk Syndromes for the assessment of ultra-high risk (UHR) criteria (identified by a prefix of capital letter plus number; 23); Schizophrenia Proneness Instrument Adult/Child & Youth version for the assessment of basic symptom criteria (no prefix) (21, 22).

b According to the notion of an independent (fourth) “impaired cognition”-dimension in psychosis that, however, is commonly defined by objective neurocognitive impairments (15, 19, 20).

APS, attenuated psychotic symptoms; BIPS, brief intermittent psychotic symptoms; COPER, basic symptom criterion “cognitive-perceptive basic symptoms”; COGDIS, basic symptom criterion “cognitive disturbances”.

### Historical Links

Although SPD as a diagnostic entity was not formulated until 1979 (24), historically, its close link to schizophrenia-spectrum psychoses was earlier established by observations on two levels (25):

- *the familial level:* observations of peculiarities resembling subthreshold features of psychosis in the (premorbid) personality of patients with schizophrenia and their biological relatives, and
- *the clinical level:* observations of patients with attenuated forms of Bleuler’s fundamental symptoms of schizophrenia without positive psychotic symptoms or severe personality deterioration.

Thus, these personality organizations were commonly called “*borderline* or *latent* schizophrenia” in the first half of the 20th century; with focus on their pathological and dysfunctional aspects (including its function as a risk indicator for psychosis), the difference between manifest psychotic disorders and their latent forms (particularly SPD) has commonly been (mis-)assumed to be one of severity or trajectory.

Both Kraepelin (26) and E. Bleuler (27) had frequently observed signs of *latent schizophrenia* in relatives of schizophrenia patients that they regarded as “essentially the same as the principle malady” (p. 234) (26) and “qualitatively identical with those of the patients themselves so that the disease appears to be only a quantitative increase of the anomalies seen in parents and siblings” (p. 238) (27). Thus, latent schizophrenia was seen as a mild expression of illness, usually not leading to help-seeking. Their and subsequent descriptions of the abnormal personality of relatives of schizophrenia patients mostly pointed towards the following core characteristics: being eccentric-odd, irritable-unreasonable, socially withdrawn, suspicious, superstitious, nervous, and hypersensitive, exhibiting an aloof and cold demeanor, functioning poorly, and speaking oddly (25).

Emphasizing the clinical link, clinical descriptions of patients emerged since the 1940s, who—though having neither familial risk nor frank schizophrenia—exhibited substantial schizophrenia-like symptoms (25). In 1953, Rado (28) coined the term *schizotype* (a contraction of “schizophrenic phenotype”; engendered by a schizophrenic genotype) to describe non-psychotic but schizophrenia-like individuals (with lifelong risk for psychotic decompensation). He assumed two major abnormalities, severe anhedonia and a distorted awareness of one’s body, from which other abnormalities would result, including a propensity for cognitive disorganization and deviant, dependent social relationships.

Building up on Rado’s ideas, Meehl (29, 30) used the term *schizotypy* to describe trait-like manifestations of *schizotaxia*, an integrative neural defect caused by a dominant *schizogene*. Relating to Bleuler (27), the core behavioral schizotypy traits were assumed to be cognitive slippage, interpersonal aversiveness (including suspiciousness and expectation of rejection due to a negative self-image of being unlovable), ambivalence, and anhedonia, with psychosis-like features merely as accessory phenomena (18, 25). In Meehl’s model, all carriers of the schizogene are schizotaxics (i.e., a true taxon of ill individuals) and—depending on environmental influences—present with graded manifestations of schizotypy, including schizophrenia as its most severe form. Consequently, *schizotaxia* (as a neural defect) and *schizotypy* (as its manifestation) equal schizophrenia-liability, while—even under the poorest environmental circumstances—a non-schizotaxic cannot become a *schizotype* or a patient with schizophrenia.

While the early schizotypy approach is aimed at commonalities with schizophrenia, the DSM-III taskforce (24) targeted the differentiation between what was to become SPD and other disorders, when formulating criteria for schizophrenia-spectrum PD. Broadly in line with this first definition, SPD is still described in DSM-5 as follows:

“a pervasive pattern of social and interpersonal deficits, including reduced capacity for close relationships; cognitive or perceptual distortions; and eccentricities of behavior, usually beginning by early adulthood but in some cases first becoming apparent in childhood and adolescence” (p. 89) (15).

Thus, unsurprisingly, SPD assessments based on this disorder-oriented view, formulate items conflating schizotypy with aspects of clinical relevance and distress (31).

### Current Perspective

Beginning with notions by Kretschmer (32) and Eysenck (33), the current understanding of schizotypy was heavily influenced by the European school of temperament and is subtly but decisively distinct from Meehl’s model (18). Proneness for psychosis was no longer believed to be a gradation of illness exclusive to a discrete subgroup of the general population but to be lying on a continuum graded throughout all people, with extreme expressions manifesting as disorders. Additionally, due to Schneider’s influential emphasis on positive symptoms (34), research on general temperaments included schizophrenia liability rather in terms of proneness for unusual perceptual experiences and magical/paranormal thinking than for Bleuler’s fundamental symptoms [e.g., Tellegen’s “Absorption” (35) or Cloninger’s “Self-Transcendence” (36)] (18).

Thus, building up further on Claridge’s work (37), schizotypy is currently not perceived as a single likely pathological dimension but as a multi-dimensional construct that is *per se* neither pathological nor equal to schizophrenia liability. Instead, at least two dimensions (positive and negative) are assumed, and it is the clustering or co-occurrence of elevated levels of them in an individual that leads to taxon-like entities like schizophrenia, SPD, or CHR (18, 38, 39). Accordingly, factor analyses of both schizotypy and SPD measures suggest that schizotypy is best understood as consisting of the same three dimensions as found in schizophrenia: a positive, a negative, and a disorganized dimension (40–42), although their conceptualization differs greatly (**Table 1**) (31, 43). Commonly and especially in the discussion of a continuum hypothesis of psychosis (44), most emphasis is put on the positive dimension, although Claridge’s fully dimensional model considers this dimension the one that is least (inherently) associated with schizophrenia liability.

### Benign Schizotypy and “Happy” Schizotypes

Thus, in contrast to the disorder-based view of schizotypy, the temperament-based models allow for the existence of benign aspects inherent to unidimensional schizotypy that, only in excess, may become pathological. This is especially true for positive schizotypy, expressing, e.g., as spiritual experiences, feelings of interconnectedness with others and/or the environment, and personal enlightenment.

The supposition that positive schizotypy and disease proneness constitute different dimensions has been argued for (implicitly but convincingly) by Claridge and colleagues (37, 45–47) who regard the difference between mentally healthy—or even “happy” (p. 255) (46)—schizotypes and schizophrenia-spectrum patients not as one of *quantity* or severity of psychosis proneness but as one of *quality* of phenomena (**Table 1**) (18). These qualitative differences are due to influences of other dimensions that are linked to negative and disorganized schizotypy (18, 38, 48, 49). Being distinct from continuously distributed schizotypy, schizophrenia is, thus, regarded as a breakdown process and endpoint on a second graded continuum that starts from SPD, making it (and other disorders) taxon-like clusters of several (individually continuous) dimensions (18).

A recent review of studies on benign schizotypy (47) concluded that high positive schizotypy in itself seems more likely to be beneficial, i.e., associated with personal wellbeing, flexible and unconventional thinking (including creativity), and favorable personality traits and psychological features (e.g., openness to experience, fantasy proneness, and spirituality). In contrast to the continuum hypothesis of psychosis focusing on positive schizotypy and in line with findings on prediction of psychoses (see below), high negative schizotypy and/or high disorganized schizotypy emerged as factors relevant to psychopathological functioning and mental ill-health (47). Lately, the view on the positive dimension was detailed by a study of the effect of schizotypy on well-being (50). Next to the different negative effects of negative and disorganized SPD features on all aspects of well-being, only the positive features suspiciousness (commonly only part of SPD but not of schizotypy assessments; **Table 1**) and ideas of reference were significantly associated with negative affect and poor environmental mastery and with poor autonomy, respectively. Other positive features, i.e., odd beliefs/magical thinking and unusual perceptual experiences, were either significantly associated with happiness, positive affect, good environmental mastery, and good personal growth, or not related to any of these outcomes (50). Notably, physical anhedonia—which is part of the negative schizotypy dimension but not of SPD—was not assessed.

## Early Detection of Psychotic Disorders

In clinical samples, the early detection of psychoses mainly follows an indicated preventive approach. Currently, a CHR state is alternatively defined by two complementary approaches (8, 51): The ultra-high risk (UHR) approach, developed to identify persons with high likelihood of transition to psychosis within the next 12 months, and the basic symptom approach, developed to detect beginning psychosis as early as possible.

The UHR criteria include the brief intermittent psychotic symptoms, the attenuated psychotic symptoms, and the “trait-state” or “genetic risk and functional decline” criterion (52, 53). The latter criterion defines the risk trait by either a first-degree family member with psychosis or by an SPD in the index patient, and the state by a functional decline. However, in clinical samples, the trait-state criterion by itself did not significantly raise risk of conversion to psychosis in recent meta-analyses (8, 54). The attenuated psychotic symptoms criterion accounts for the vast majority of CHR patients. Attenuated psychotic symptoms are mainly defined by sub-threshold psychotic-like experiences (as earlier defined on a clinical continuum by the Chapmans) (55) and by positive features of SPD (13). Nevertheless, attenuated psychotic symptoms differ from corresponding trait-like features of SPD (and paranoid PD) by their obligate recent onset or worsening (**Table 1**); i.e., by capturing early state-like signs of an emerging disorder that allow the initiation of an indicated prevention (13, 56). The trait-state distinction between positive schizotypy and APS was recently supported in a study showing significant changes in APS but not positive schizotypy over 1 year (57).

The basic symptom criteria include “cognitive disturbances” and the “cognitive-perceptive basic symptoms” (58, 59). Of these, the latter lacked sufficient meta-analytical evidence to be already recommended for clinical practice (7). Contrary to the trait character of schizotypy and SPD, basic symptoms decidedly have state character, as, by definition, they differ from what patients consider to be their “normal” mental self (57, 59, 60). Basic symptoms are conceptualized as the earliest primary psychopathological correlates of the neurophysiological disturbances of information processing underlying the development of attenuated and frank psychotic symptoms, which develop based on and partly in reaction to basic symptoms (61, 62). Thus, independently of any thought content or perception, basic symptoms are disturbances in mental processes themselves, thereby clearly differing from more content-related positive features of schizotypy and SPD, and attenuated and brief limited psychotic symptoms (**Table 1**) (60–62).

Studies of personality dimensions, schizotypy, PDs, and SPD, in CHR samples indicate the following:

- CHR patients, compared to CHR-negative patients, are more often high scorers on all four higher-order personality dimensions simultaneously, i.e. emotional dysfunction, inhibitedness, dissocial behavior, and compulsivity (63), rather than exhibiting a distinct “psychosis profile,” e.g., of high neuroticism, low extraversion, and medium agreeableness and conscientiousness (64).
- Studies using positive and negative schizotypy assessments, such as the four Wisconsin Schizotypy Scales (65, 66), suggest that pronounced physical anhedonia enhances risk for psychosis, though likely only in the presence of CHR states (67, 68); moreover, physical anhedonia also predicted presence of UHR but not of basic symptom criteria (67).
- Studies using SPD assessments, such as the Schizotypal Personality Questionnaire (66, 69), in CHR patients indicated that SPD, particularly (paranoid) ideas of reference and lack of close friends, predicted psychosis (13) and that SPD assessment might help to identify CHR patients, especially those meeting the trait-state UHR criterion (70).
- When other PD were simultaneously considered, schizoid rather than schizotypal personality traits predicted conversion to psychosis in CHR patient, mainly by deficits in social interaction (that are also partly included in schizotypy assessments of social anhedonia) but not by indifference and emotional coldness (56).

Furthermore, in clinical samples defined by schizotypal disorder, schizoid PD or SPD, up to 48% developed psychosis, which was best predicted by unusual or paranoid ideas and social isolation (13). A similar pattern of predictors was found in non-clinical genetic-risk and community samples, in which positive schizotypy and SPD assessments of unusual and paranoid ideas and unusual perceptual experiences were main predictors of psychoses, whereby social or physical anhedonia and social withdrawal further improved prediction of psychosis—but even more of schizophrenia-spectrum disorders—in some studies (13, 71). Thus, schizotypy and SDP features seem to detect psychosis early; yet, the psychosis-predictive power of single assessments seems to depend on the examined population and, likely, on the interplay between positive and negative dimensions (49). Additionally, the inherent conflation of schizotypy features with distress found in inventories based on Meehl and the SPD conceptualization must be kept in mind (31).

Furthermore, it must be observed that little is known about the role of the disorganized dimension that has hardly been studied. Thus, some effects might be misattributed to the positive and negative schizotypy dimension, as recently shown for the earlier likely misattributed association of negative affective with positive schizotypy that is better explained by one with disorganized schizotypy (72, 73).

## Early Detection of Severe Schizophrenia-Spectrum Personality Disorder

Although schizotypal disorder and SPD have been studied for their propensity to predict psychosis in several studies (13), few studies have examined their predictors. An early study followed children clinically diagnosed as “schizoid” over a mean course of 18 years, whereby “schizoid” was defined by solitude, impaired empathy/emotional detachment, mental rigidity, hypersensitivity with a tendency to paranoid ideas, and odd communication (74). At follow-up, three quarters had developed SPD and 8% psychosis; only 13% had clearly recovered from their schizoid symptoms (75). Moderate stability of the three SPD dimensions across adolescence, i.e., from age 11 to age16, along with a clear indication of their heritability (*h*² = 38–57%) (76) at each assessment time has also been reported (77). Variance in SPD assessment scores at 16 years could be decomposed in 36% stable genetic, 3% stable environmental, 42% time-specific genetic, and 19% time-specific environmental influences, with the positive dimension score being explained by genetic variance only at age 11 years. SPD usually begins by early adulthood, and only rarely in childhood and adolescence (15). Furthermore, an increase in schizotypy and SPD severity across adolescence with a subsequent decrease in adulthood was repeatedly reported (78, 79). Thus, particularly when in concert with a parental schizophrenia-spectrum disorder, pronounced persistent or increasing schizotypy features (49) might currently be the best predictors of adult SPD in youth, especially when of the negative socially impaired and the positive paranoid-suspicious kind.

Other clinical (e.g., heightened anxiety levels), environmental (e.g., childhood adversity and trauma), genetic (e.g., Val allele of the Val^158^Met COMT polymorphism), neurobiological (e.g., various brain abnormalities in frontal, temporal, striatal, and parahippocampal regions), social-cognitive (e.g., poor emotion recognition), and neuropsychological (e.g., jumping-to-conclusion) risk factors of SPD resemble those described for schizophrenia (80, 81), thus not displaying a unique pattern that could be used for its prediction specifically.

## Conclusions

We conclude that schizotypy, SPD (and likely other schizophrenia-spectrum PD), and psychotic disorder are rather manifestations of discrete profiles (i.e., qualitatively distinct taxon-like clusters) of schizotypy or SPD dimensions than merely states of different severity on only one dimension. In doing so, positive schizotypy features—other than the distressing SPD feature of paranoid ideas of reference and suspiciousness—do not appear to be pathognomonic by themselves. This is in contrast to continuum models of psychosis that mainly rely on positive features and assume a progression from positive schizotypy and SPD traits *via* psychotic-like experiences and attenuated psychotic symptoms to psychotic positive symptoms and, finally, schizophrenia (44). Pathological personality processes rather seem to require an interaction of the positive dimension with the negative and/or disorganized dimension, at which, of the positive features, trait-like distressing paranoid ideas of reference and suspiciousness, which are unique to the positive SPD dimension, seem to be most relevant and a starting point on the suggested SPD-psychosis continuum that is distinct from the potentially benign positive schizotypy dimension. The SPD-psychosis continuum, however, likely also involves state-like subclinical positive symptoms such as UHR symptoms that are predictive of psychosis. In doing so, the trait or state character of the positive features might be crucial for the development of SPD or psychosis in late adolescence or young adulthood.

In light of the merits of early diagnosis, a differential early detection of incipient psychotic disorders and schizophrenia-spectrum PD, guided by a comprehensive assessment of all relevant schizotypy-SPD-psychosis dimensions, is necessary—also in light of calls for dimensional diagnostic systems (82), yet requires more research into their differential prediction.

## Author Contributions

FS-L was responsible for the conception of the work and drafted the first versions of this work. PG and IN revised it critically for important intellectual contents. All authors provided approval for publication of the content.

## Conflict of Interest Statement

The authors declare that the research was conducted in the absence of any commercial or financial relationships that could be construed as a potential conflict of interest.
